# Central Angiotensin II Stimulation Promotes β Amyloid Production in Sprague Dawley Rats

**DOI:** 10.1371/journal.pone.0016037

**Published:** 2011-01-28

**Authors:** Donglin Zhu, Jingping Shi, Yingdong Zhang, Bianrong Wang, Wei Liu, Zhicong Chen, Qiang Tong

**Affiliations:** 1 Department of Neurology, Nanjing Brain Hospital, Nanjing Medical University, Nanjing, People's Republic of China; 2 Department of Neurology, School of Medicine, Nanjing University, Nanjing, People's Republic of China; Johns Hopkins, United States of America

## Abstract

**Background:**

Stress and various stress hormones, including catecholamines and glucocorticoids, have recently been implicated in the pathogenesis of Alzheimer's disease (AD), which represents the greatest unresolved medical challenge in neurology. Angiotensin receptor blockers have shown benefits in AD and prone-to-AD animals. However, the mechanisms responsible for their efficacy remain unknown, and no studies have directly addressed the role of central angiotensin II (Ang II), a fundamental stress hormone, in the pathogenesis of AD. The present study focused on the role of central Ang II in amyloidogenesis, the critical process in AD neuropathology, and aimed to provide direct evidence for the role of this stress hormone in the pathogenesis of AD.

**Methodology/Principal Findings:**

Increased central Ang II levels during stress response were modeled by intracerebroventricular (ICV) administration of graded doses of Ang II (6 ng/hr low dose, 60 ng/hr medium dose, and 600 ng/hr high dose, all delivered at a rate of 0.25 µl/hr) to male Sprague Dawley rats (280–310 g) via osmotic pumps. After 1 week of continuous Ang II infusion, the stimulation of Ang II type 1 receptors was accompanied by the modulation of amyloid precursor protein, α-, β-and γ-secretase, and increased β amyloid production. These effects could be completely abolished by concomitant ICV infusion of losartan, indicating that central Ang II played a causative role in these alterations.

**Conclusions/Significance:**

Central Ang II is essential to the stress response, and the results of this study suggest that increased central Ang II levels play an important role in amyloidogenesis during stress, and that central Ang II-directed stress prevention and treatment might represent a novel anti-AD strategy.

## Introduction

Alzheimer's disease (AD) is the most common neurodegenerative disorder worldwide [1]. It is characterized neuropathologically by the formation of senile plaques and neurofibrillary tangles, and clinically by the progressive deterioration of memory and other cognitive functions [2], which cannot be prevented using currently available treatments [3]. Senile plaques are primarily composed of a 40–42-amino acid peptide denoted as β amyloid (Aβ), which is derived from sequential cleavage of amyloid precursor peptide (APP) by β-secretase and γ-secretase (the amyloidogenesis pathway) [1].The accumulation and deposition of Aβ in selective brain regions is a major cause of neurotoxicity and is assumed to be a culprit for inducing the pathologic processes of AD [4]. However, APP can also be processed by an α–γ secretase stepwise pathway, which precludes the generation of Aβ (the antiamyloidogenesis pathway).

Several years after the publication of descriptive reports of α-secretase, three responsible enzymes, belonging to the A Disintegrin And Metalloproteinase (ADAM) family, have been identified with α-secretase activity: ADAM 9, ADAM 10, and ADAM 17[5]. Moreover, a recent knockdown study confirmed that ADAM 10, but surprisingly not ADAM 9 or ADAM 17, was the physiologically relevant, constitutive α-secretase of APP [6]. The identity of β-secretase has also been studied, and a novel aspartyl protease, named the β-site APP-cleaving enzyme (BACE1), was revealed in five independent reports [7]–[11]. BACE1 cleavage of APP has subsequently been proven to be a prerequisite for Aβ generation [12]. γ-secretase is a membrane-embedded protease complex consisting of presenilin 1 (PS1), nicastrin, anterior pharynx-defective 1, and presenilin enhancer 2 [13]. γ-secretase specificity determines the ratio of two peptide products, Aβ_40_ and the more neurotoxic Aβ_42_, and mutations in its catalytic subunit PS1, which lead to an increased Aβ_42_/Aβ_40_ ratio, account for most cases of genetic AD [14]. However, because genetic AD accounts for only a small percentage (probably less than 1%) of the total number of cases [15], the development of sporadic AD is still linked to environmental influences. Stress, particularly chronic adverse stress, is one of the environmental factors that has been suggested to contribute to the onset and progression of AD [16]. The stress response is often associated with high levels of stress hormones, such as catecholamines (CAs, effectors of the sympathetic adrenomedullary system), glucocorticoids (GCs, effectors of the hypothalamic pituitary adrenal axis), and their regulator, central angiotensin II (Ang II) [17]. Both CAs [18], [19] and GCs [20], [21] are known to be linked to the pathogenesis of AD, but their regulator, central Ang II, deserves further consideration.

Central Ang II, by binding the Ang II type 1 receptor (AT_1_R) at all key hypothalamic regulatory centers or higher centers, plays a critical role in the activation of the sympathetic adrenomedullary system and hypothalamic pituitary adrenal axis, as well as in the secretion of CAs and GCs [17], [22]. Central Ang II has thus been accepted as a fundamental stress hormone in the body [17], [23]. However, central Ang II has also been implicated in several central nervous system disorders. For example, hippocampal Ang II-immunopositive neurons with distorted processes were detected in Aβ deposits in AD brains [24], and enhanced immunoreactivity of Ang II, AT_1_R, and angiotensin-converting enzyme (ACE) was found in a postmortem examination of patients with AD [25]. Moreover, some studies also showed that Ang II could inhibit potassium-mediated release of acetylcholine [26], [27], induce oxidative stress[28], activation of mitogen-activated protein kinases (MAPKs) [29], and inflammation [30], and was involved in blood–brain barrier maintenance [31] and cell survival [32], all of which are relevant to AD [32]–[35]. When administered to AD and prone-to-AD animals, some Ang II receptor blockers (ARBs), such as losartan, valsartan, candesartan, telmisartan, and olmesartan, were associated with improved cognitive impairment and/or attenuated Aβ production [36]–[40]. It is therefore vitally important to determine the potential role of Ang II in AD, especially in amyloidogenesis, given the proven involvement of stress and stress hormones in the pathogenesis of AD. The present study therefore used a rat model of AD to provide direct evidence for the role of Ang II in amyloidogenesis and the pathogenesis of the disease.

## Results

### Central Ang II Stimulation Caused a Dose-dependent Increase in AT_1_R expression

**Figure 1:**
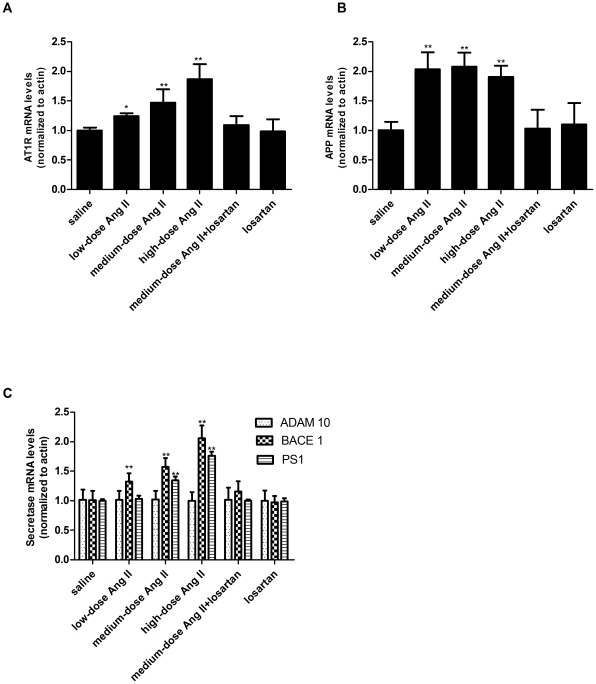
Expression assays for AT_1_R and APP processing pattern by qPCR. Expression analysis showing the normalized mRNA levels of (**A**) AT_1_R, (**B**) APP, (**C**) ADAM 10, BACE1 and PS1. n = 7 for AT1R and n = 6 for all others. CTs (threshold cycles) were used as the readout. The results were obtained using the comparative Ct method and the arithmetic formula 2^−ΔΔCt^_._ Data were normalized to β-actin and represented as fold increases over saline controls. *p<0.05 or **p<0.01 versus the control group receiving saline infusion.

**Figure 2:**
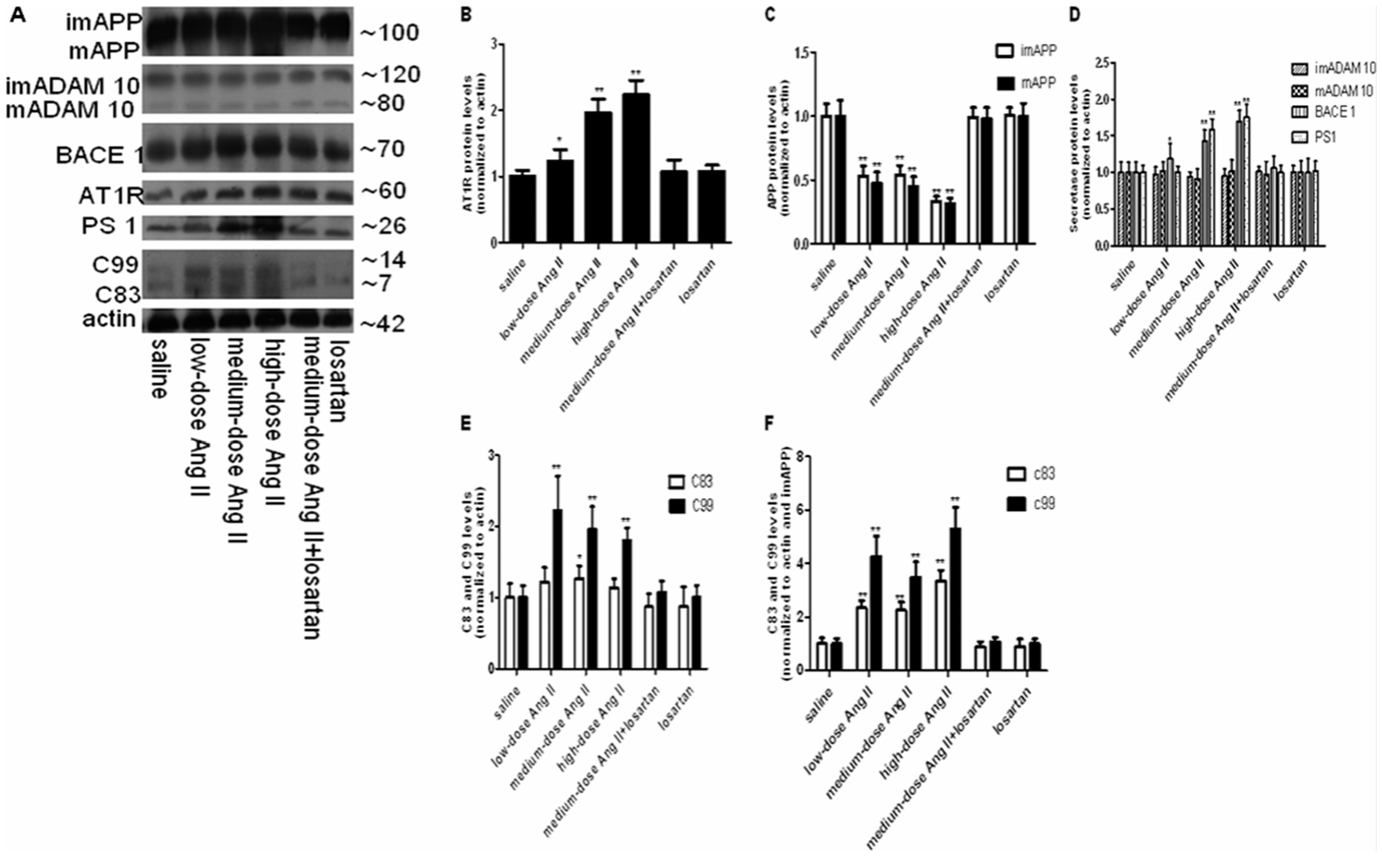
Expression assays for AT_1_R and APP processing pattern by WB. The same gels were blotted for β-actin as an internal standard. n = 6 for each group. (**A**) Representative enhanced chemiluminescence radiographs of immunoblots showing the immunoactivities of AT_1_R, APP, ADAM 10, BACE1, PS1, C83, C99 and β-actin. Density analysis showing levels of (**B**) AT_1_R, (**C**) APP, (**D**) ADAM 10, BACE1, and PS1, (**E**) (**F**) C83 and C99. Data in (**B–E**) were normalized by dividing the density of the AT_1_R, ADAM 10, BACE1, PS1, C83 and C99 bands by the density of the β-actin band. Data in (**F**) were normalized by dividing the density of the C83 and C99 band by that of the β-actin band and then the imAPP band. All data were represented as fold increases over the saline controls. *p<0.05 or **p<0.01 versus the control group receiving saline infusion. imAPP, immature APP; mAPP, mature APP; imADAM 10, immature ADAM 10;mADAM 10, mature ADAM 10.

AT_1_R mRNA and protein levels were assayed using quantitative polymerase chain reaction (qPCR) and western blotting (WB), respectively, after intracerebroventricular (ICV) administration of Ang II. A dose-dependent increase in AT_1_R mRNA levels was observed 1 week after central infusion of graded doses of Ang II. An almost 1.5-fold increase was observed following medium-dose Ang II stimulation, which was attenuated by combination with losartan. Losartan infusion alone had no effect on AT1R mRNA (p>0.05) (**Figure 1A**). Similar changes in AT_1_R protein levels were identified by WB (**Figure 2A and 2B**).

These results suggest that central AT_1_R expression could be dose-dependently elevated by increased central Ang II, as confirmed by concomitant losartan administration, and that baseline AT_1_R expression was unaffected by ARBs, in the absence of stimulation by specific ligands.

### Central Ang II Stimulation Downregulated Full-length APP Protein but Upregulated its mRNA

Full-length APP protein levels were analyzed using a carboxyl-terminal-directed antibody that recognizes the carboxyl terminal fragments (CTFs) and the full-length APP holoproteins (mature APP or mAPP and immature APP or imAPP). The binding capacity of the antibody has been confirmed in previous studies [41]–[43]. WB showed a remarkable reduction in both mature and immature APP holoprotein levels in central Ang II-treated animals: about a 50% reduction with low-dose and medium-dose Ang II (p<0.01), and about a 70% reduction with high-dose Ang II (p<0.01), relative to saline-treated controls (**Figure** **2A** **and** **Figure** **2C**). APP transcription analyzed by qPCR showed an almost 2-fold increase in all Ang II-treated animals (p<0.01) (**Figure** **1B**). These effects were abolished by concomitant losartan treatment, and could therefore be attributed to Ang II.

### Central Ang II Stimulation Had Different Effects on Secretase Expression

mRNA and protein levels of ADAM 10, BACE 1, and PS1, representing the expression levels of α-, β-, and γ-secretase, were also evaluated. As shown in **Figure 1C** **,** **Figure 2A** and **Figure** **2D**, ADAM 10 mRNA and protein remained at baseline levels after infusion of central Ang II or its blockers for 1 week(p>0.05), implying that central Ang II stimulation failed to affect ADAM 10 expression.

However, BACE1 mRNA, assessed by qPCR, was increased 1.3-fold following low-dose (p<0.01), and 1.6-fold following medium-dose Ang II treatment (p<0.01), and almost 2.1-fold following high-dose Ang II treatment (p<0.01) (**Figure** **1C**). BACE1 protein levels assessed by WB also showed dose-dependent increases in the Ang II-treated groups (**Figure** **2A and 2D**). The marked increases in BACE1 mRNA and protein levels following medium-dose Ang II treatment were normalized by losartan(p>0.05), implying that these alterations were associated with central Ang II stimulation.

Regarding PS1 levels, low-dose Ang II infusion had no effect on PS1 gene expression (p>0.05), whereas medium-dose and high-dose Ang II infusions were followed by a significant increase (p<0.01), and an even higher increase (p<0.01), respectively. These increases were cancelled by blockade of AT_1_R with losartan (**Figure 1C**), suggesting that they were induced by stimulation of AT_1_R. PS1 protein expression analyzed by WB showed similar changes to those demonstrated for its gene expression.

### Central Ang II Stimulation Had Different Effects on Secretase Activity

The activities of α, β-, and γ-secretase were evaluated by measuring the fluorescence intensity after incubation with a synthetic fluorescent peptide substrate.

**Figure 3:**
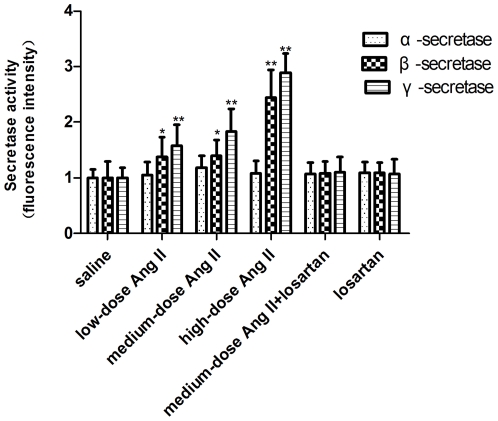
Activity assays for α-, β- and γ-secretase. Activity analysis showing activity levels of α-, β-and γ-secretase. Data are represented as fold increases over saline controls. n = 5 for each group. All samples were analyzed in duplicate. *p<0.05 or **p<0.01 versus the control group receiving saline infusion.

As shown in **Figure 3**, no significant alterations in α-secretase activity were shown in any Ang II-treated animals(p>0.05), suggesting that central Ang II had no effect on the activity of α-secretase.

β-secretase activity was increased less than 1.4-fold following both low-dose and medium-dose Ang II(p<0.05), but by almost 2.5-fold by high-dose Ang II(p<0.01) (**Figure** **3**). Then, enhanced β-secretase activity was blocked by losartan and disappeared in single losartan, suggesting that the enhanced activity was due to Ang II.

The results of activity assay for γ-secretase are shown in **Figure** **3**. Treatment with graded doses of Ang II was followed by an approximately 1.6–1.8–2.9-fold stepwise increase in γ-secretase activity, with no significant difference in activities between low-dose and medium-dose Ang II treatment (p>0.05). The increase following medium-dose Ang II treatment was normalized by concomitant losartan treatment (p>0.05), supporting the causative role of Ang II in these changes.

### Central Ang II Stimulation Caused Significant Alterations in the Levels of APP α-CTF (C83) and β-CTF (C99), Concentrations of Aβ_40_ and Aβ_42_, and Aβ_42_/Aβ_40_ Ratios

Cleavage of APP by α- or β-secretase results in the production of APP CTFs, i.e., C83 and C99, the latter of which is the direct substrate of γ-secretase and immediate precursor of Aβ[44]. As mentioned above, the antibody that recognizes full-length APP can also be used to detect its CTFs, C83 and C99.

When normalized to β-actin, WB of C83 showed an almost 1.3-fold increase following medium-dose Ang II treatment (p<0.05), but no changes in response to other doses of Ang II (p>0.05) (**Figure 2A and 2E**). Simultaneous WB of C99 showed an approximately 2.0-fold increase following low-dose and medium-dose Ang II treatments (p<0.01), but a 1.8-fold increase following high-dose Ang II treatment (p<0.01) (**Figure 2A and 2E**). The productions of C83 and C99 were represented after normalization to both β-actin and APP. As shown in **Figure 2F**, when normalized to β-actin and then immature APP (imAPP), C83 production showed a 2.3-fold increase following low-dose and medium-dose Ang II (p<0.01), and a higher 3.3-fold increase following high-dose Ang II treatment (p<0.01). C99 production showed more significant increases, including a 4.3-fold increase in response to low-dose Ang II (p<0.01), a 3.5-fold increase following medium-dose Ang II (p<0.01), and a 5.3-fold increase following high-dose Ang II (p<0.01).

**Figure 4:**
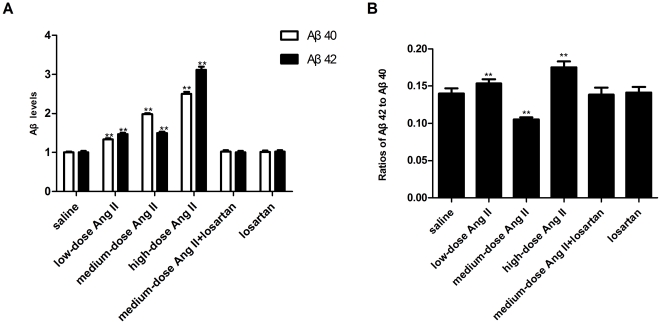
Production assays for Aβ_40_ and Aβ_42_ by ELISA. (**A**) Concentrations of Aβ_40_ and Aβ_42_ in rat cortices. Data are represented as fold increases over saline controls. n = 6 for each group. All samples were analyzed in duplicate. (**B**) Aβ_42_/Aβ_40_ ratios. Data were calculated by dividing the Aβ_42_ concentration by the Aβ_40_ in the same sample. *p<0.05 or **p<0.01 versus the control group receiving saline infusion.

The concentration of Aβ in the rat cerebral cortices was analyzed by sandwich ELISA. As shown in **Figure 4A** and **Figure S1,** low-dose Ang II treatment produced a 1.3-fold increase in Aβ_40_ concentration (from 30.26±0.56 in controls to 40.38±0.70, p<0.01), but a 1.5-fold increase in Aβ_42_ concentration (from 4.23±0.17 in controls to 6.20±0.14, p<0.01). When the dose of Ang II was increased by 10-fold, the increase in Aβ_40_ increased by almost 2-fold (to 59.98±0.84, p<0.01), but no further significant increase in Aβ_42_ concentration occurred (6.31±0.16). A further 10-fold increase in Ang II resulted in increases in Aβ_40_ and Aβ_42_ concentrations of nearly 2.5-fold (to 75.48±1.89, p<0.01) and 3-fold (to 13.20±0.32, p<0.01), respectively. These effects did not occur following the administration of concomitant or single losartan (p>0.05).

We also calculated the Aβ_42_/Aβ_40_ ratios in the same samples. As shown in **Figure 4B**, the ratio was approximately 0.140±0.007 in the controls animals, and this increased to 0.154±0.005 following low-dose Ang II treatment (p<0.01), but decreased to 0.105±0.003 following medium-dose Ang II treatment (p<0.01). After a rebound increase to 0.175±0.008 following treatment with high-dose Ang II (p<0.01), the ratios returned 0.138±0.010 and 0.141±0.008 with concomitant or single losartan treatment (p>0.05).

The effects of medium-dose Ang II on C83 and C99 levels, concentrations of Aβ_40_ and Aβ_42_, and on the Aβ_42_/Aβ_40_ ratios were attenuated or even abolished by blocking AT_1_R signaling, confirming that these effects resulted from central Ang II binding to AT_1_R.

## Discussion

Ang II has multiple tasks in the brain and plays key roles in the regulation of blood pressure, fluid homeostasis, cerebral blood flow, and the response to stress [45]. These classical, well-defined actions of Ang II in the brain are mediated by stimulation of the AT_1_R [45]. Stress enhances the production of peripheral and brain Ang II [46]–[48] and the expression of peripheral and brain AT_1_R [46], [49]–[51]. Ang II does not readily cross the blood–brain barrier, and the present study therefore used ICV Ang II administration in rats to model the increased central Ang II levels occurring during the stress response. Both gene and protein levels of AT_1_R were dose-dependently upregulated after 1 week of ICV Ang II infusion, suggesting that self-upregulation of AT_1_R expression occurred after stimulation by Ang II, in accordance with the results of previous studies [52]–[55]. The regulation of AT_1_R expression and stimulation is important, as it determines the level of activation of the brain Ang II system [22].

APP is the source of Aβ. In the present study, graded doses of central Ang II caused a 50–70% reduction in the level of APP protein, which did not correspond to the more significant increases in the production of C83, C99, and Aβ. This discrepancy implies that APP expression and processing were enhanced by central Ang II, which was further supported by the upregulation of APP mRNA levels.

APP is predominately cleaved by α-secretase under physiologic conditions [56]. In the current study, central Ang II had no effect on ADAM 10 protein or mRNA levels or activity. However, although α-secretase expression was represented by the level of ADAM 10 in this study, other members of the ADAM family, including ADAM 9 and ADAM 17, have also been proposed to display α-secretase activities [5]. Even a close homolog of BACE1, BACE2, has been found to be responsible for α-secretase-like proteolytic cleavage of APP [57], [58]. Thus the regulation of other putative α-secretase enzymes by Ang II should also be assayed.

BACE1 is the initiating and putatively rate-limiting enzyme in Aβ generation [59]. Levels of BACE1 mRNA and protein were elevated by Ang II in the current study. Previous studies found that the BACE1 promoter contains a number of putative transcription factor-binding sites, including nuclear factor kappa-light-chain-enhancer of activated B cells (NFκB) [60], activator protein 1 (AP 1) [61] and cAMP response element-binding protein (CREB) [61]. Activation of these transcription factors is involved in Ang II signaling [52], [55], [62], and this mechanism may therefore provide a basis for Ang II-induced BACE1 expression. Significant increases in β-secretase activity were also observed in this study. Oxidative stress has recently been identified as a factor involved in the stimulation of β-secretase activity [63], [64], and is also well implicated in Ang II signaling [28]. The stimulation of β-secretase might thus result from Ang II-mediated oxidative stress. Several reports have reported BACE1 to be a stress-response protein, or β-secretase to be a stress-induced protease, because BACE1 expression and activity were elevated following acute or chronic stress [59]. This might suggest a potential link between stress-induced Ang II and altered BACE1 expression and activity.

The lack of α-secretase stimulation was expected to be reflected in a lack of C83 production, while significant increases in BACE1 expression and activity were expected to be associated with proportionate changes in C99 production. However, when normalized to β-actin, an almost 1.3-fold increase in C83 levels was detected following medium-dose Ang II administration, though low-dose and high-dose Ang II had no effects, and high-dose Ang II resulted in an almost 1.8-fold increase in C99 levels, while low-dose and medium-dose Ang II produced 2-fold increases. This inconsistency between C99 levels and significant alterations in BACE1 suggests that C99 was short-lived. On the other hand, the uncoupling of α-secretase activity and C83 levels might imply the existence of an additional mechanism for the production of C83. Recent studies have reported that C99 could be converted to C83 by α-secretase [6], [65], which could account for the C83 levels observed in the current study. This possibility is further supported by the sharp increases in C83 levels and the opposite effects of medium-dose Ang II on C83 and C99, after normalization to both β-actin and APP.

PS1 is a key determinant of γ-secretase activity [66]. We analyzed the expression of PS1 and found that stimulation of the AT_1_R by medium-dose or high-dose Ang II was accompanied by increased PS1 expression, though these changes were not observed after low-dose Ang II infusion. As a γ-secretase catalytic subunit, increased PS1 expression has often been found to occur simultaneously with augmented γ-secretase activity [67], while repression of PS1 transcription inhibited γ-secretase mediated processing of APP [68]. However, overexpression of PS1 was not sufficient to generate increased γ-secretase activity or produce more products in previous studies[69], suggesting that PS1 does not act alone. Given the complicated situation, γ-secretase enzymatic activity in this study was evaluated in homogenates *in toto*, and equivalent enhancements were observed following low-dose and medium-dose Ang II treatment, with greater enhancement after high-dose treatment. Although the current study was unable to elucidate the precise mechanism of AT_1_R regulation of γ-secretase, several lines of evidence have indicated possible mechanisms, as follows: central Ang II is well known to induce the activation of some transcription factors, such as AP 1 [52], NFκB [55] and CREB [62], which have been localized in PS1 promoter and implicated in PS1 gene expression [67], [70]–[72]. Ang II could also induce the activation of several MAPK members, which have been associated with enhanced γ-secretase activity [73]. These results imply that activation of AT_1_R could promote PS1 expression through the activation of transcription factors, and could mediate the direct phosphorylation of PS1 or γ-secretase at specific sites by targeting the MAPK pathway. Second, recent studies have shown that CAs can enhance γ-secretase activity by inducing autophagy [18], and because central Ang II promotes CA utilization [74], this may represent an indirect pathway whereby central Ang II can regulate γ-secretase activity. However, the study indicating that CAs promote amyloidogenesis by inducing autophagy was performed *in vitro* [18], and further research is needed to confirm these results *in vivo*.

Changes in the processing pathway of APP resulted in increased Aβ production; a dose-dependent increase in secreted Aβ_40_ concentration, and a 1.5–3-fold increase in Aβ_42_ concentration were achieved following Ang II stimulation. Notably, the Aβ_42_/Aβ_40_ ratios were altered. This ratio was linked to specificity of γ-secretase in previous studies [75], [76], implying that Ang II could cause shifts in APP amyloidogenic processing. Furthermore, the neurotoxicity of Aβ was recently shown to be associated with small changes in the Aβ_42_/Aβ_40_ ratio [77], and Ang II-induced shifts in this ratio should thus be taken into serious consideration. However, losartan given together with medium-dose Ang II abolished all effects of Ang II on APP, the three secretases, and Aβ. These findings suggest that the actions of central Ang II in amyloidogenesis are mediated by activation of the AT1R.

Hypertension has been identified as a strong risk factor for AD in epidemiological studies [78], [79]. ICV infusion of Ang II in the present study might have resulted in an increase in blood pressure (BP) in the rats. Furthermore, previous studies in both animals and humans found that ARBs helped to preserve cognitive function through a mechanism independent of their antihypertensive effects [80]–[83]. The effects of systemic BP were outside the scope of the current study. However, AD has recently been proposed to be a vascular disease, rather than a neurodegenerative disease [84], [85]. Because Ang II is a potent vasoactive peptide, stress-induced and Ang II-involved hypertension may contribute to amyloidogenesis. Thus further studies are needed to investigate the link between hypertension, especially nonpharmacologic hypertension, and amyloidogenesis.

In conclusion, the results of this study demonstrated that central Ang II potentiated amyloidogenesis *in vivo*, through modulation of various components of the APP processing pathway; these actions could be completely abolished by concomitant ICV infusion of losartan. This study not only provides direct etiological evidence for central Ang II, a fundamental stress hormone, in the pathogenesis of AD, but also suggests that ARBs, already used in clinical settings, may be potentially effective agents for the prevention and treatment of AD.

## Materials and Methods

### Reagents and preparation

Ang II (Sigma) was dissolved in sterile saline to 24 ng/µl, 240 ng/µl, and 2,400 ng/µl, as adapted from a recent study [86]. Losartan (Sigma) was added to 240 ng/µl Ang II to a concentration of 800 ng/µl, which was adequate to abolish the effects of 240 ng/µl Ang II *in vivo*, as verified in our preliminary experiments.

Osmotic pumps (Alzet, model 2004) with a brain infusion kit (Alzet) were filled with reagents or sterile saline and placed into sterile saline at 37°C for at least 40 hours, according to the manufacturer's instructions. The pumps were designed to work at a rate of 0.25 µl/hr. The actual doses of Ang II delivered into rat brains were thus 6 ng/hr, 60 ng/hr and 600 ng/hr.

### Animals and surgery

Male Sprague Dawley rats (from the National Rodent Laboratory Animal Resources Shanghai branch of China) weighing 280–310 g were housed in a controlled environment at 25±2°C with alternating 12 hr light and dark cycles. They were provided with food and water *ad libitum*. All rats were acclimatized in our animal facility for at least 1 week before the experiments were conducted. Animal care and experiment protocols were in accordance with the Guide for the Care and Use of Laboratory Animals of Nanjing Medical University and were approved by the Biological Research Ethics Committee of Nanjing Medical University (permit number: NYLL2009-0005). All surgery was performed under anesthesia and all efforts were made to minimize the suffering of the animals.

A total of 54 rats were randomly divided into six groups, according to the materials in the pumps: sterile saline; low-dose Ang II; medium-dose Ang II; high-dose Ang II; medium-dose Ang II + losartan; and losartan. Animals in each group were subjected to ICV administration via a pump for 7 days. Pump implantation was carried out using a well-established procedure in our department [87]. In brief, animals were anaesthetized by intraperitoneal injection of 10% chloral hydrate (0.35 ml/100 g). Anaesthetized rats were placed in a stereotactic frame (David Kopf), and the skull was exposed under sterile surgical conditions. Stainless steel cannulas from the brain infusion kit were implanted in the right lateral ventricle, relation to the bregma, at: 1.4 mm lateral and 0.8 mm posterior on the right. A right-sided burr-hole was drilled in the skull, 3.5 mm below the top of the cortex. The pumps were then installed in subcutaneous pockets on the lateral dorsum of the rats. Dental cement was used to affix the catheters to the skull, and the wounds were closed with sutures. Body temperature was monitored throughout the procedure using a rectal temperature probe and maintained at 37±0.5°C with a thermostatically-controlled infrared lamp.

After 7 days, anaesthetized animals were sacrificed by decapitation and brains were immediately dissected on ice, frozen in liquid nitrogen, and maintained at −80°C until assayed.

### Total RNA extraction, reverse transcription (RT) and qPCR assay of gene expression

Cortices were homogenized in 10 volumes of TRIzol reagent (Invitrogen) and total RNA was extracted, according to the manufacturer's instructions. The quantity and quality of the resulting RNA were validated using a spectrophotometer (Hitachi). Of the total RNA, 500 ng was reverse transcribed to cDNA using a RT reagent kit (Takara) at 37°C for 15 minutes, followed by 85°C for 5 seconds, according to the manufacturer's instructions. The products were then stored at −20°C until use. For quantitative SYBR Green (2×SYBR premix Ex Taq, Takara) real-time PCR, equal amounts of cDNA were used for each reaction. Primer sequences were designed using primer 3 software as follows:

murine β-actin: 5′-AGCTATGAGCTGCCTGACGGC-3′ and 5′-CATGGATGCCACAGGATTCCA-3′;

murine AT1R: 5′-CCTCTACGCCAGTGTGTTCC-3′ and 5′-GCCAAGCCAGCCATCAGC-3′;

murine APP: 5′-GTGAGCGACGCCCTTCTCGTGCC-3′ and 5′-TCCGTGTCAAAGTTGTTCCTGTTGC-3′;

murine ADAM 10: 5′-GCCTATGTCTTCACGGACCG-3′ and 5′-TGCCAGACCAAGAACACCAT-3′;

murine BACE 1:5′-CATTGCCATCACTGAAT-3′ and 5′-CAGTGCCTCAGTCTGGTTGA-3′;

murine PS1: 5′-GAGGAAGACGAAGAGCTGACAT-3′and 5′-GAAGCTGACTGACTTGATGGTG-3′.

A two-step qPCR was performed using an ABI 7500 Real-Time PCR System (Applied Biosystems). After 30 seconds of initial denaturation at 95°C, the thermal profile included 40 two-step cycles: step 1 for denaturation at 95°C for 5 seconds and step 2 for annealing and extension at 60°C for 34 seconds. Data were collected at step 2. The results were obtained using the comparative Ct method and the arithmetic formula 2^−ΔΔCt^.

### Tissue extraction for protein- and peptide-directed assays

To extract total protein, cortices were placed in 10 volumes of ice cold RIPA buffer (50 mmol/l Tris-HCL, 150 mmol/l sodium chloride, 1% Triton X-100, 1% sodium deoxycholate, 0.1% sodium dodecyl sulfate) supplemented with protease inhibitors (Sigma). The tissue was homogenized for 10 s in an ultrasound homogenizer (IKA Laboratory). Tissue homogenates were then centrifuged for 15 min at 12,500 *g* at 4°C (Beckman). Supernatants were carefully collected.

For Aβ concentration assays, tissue extracts were prepared as described previously [88], with some modifications. Cortical tissues were homogenized in 10 volumes of high-salt buffer (100 mmol/l Tris-HCL, pH 7.0, 1 mmol/l EGTA, 0.5 mmol/l magnesium sulfate_,_ 750 mmol/l sodium chloride, 20 mmol/l sodium fluoride) containing protease inhibitors (Sigma). Soluble Aβ was extracted using diethyl acetate 0.4% in 100 mmol/L sodium chloride and the sample was centrifuged for 60 min at 100,000 *g* at 4°C (Beckman). The supernatants were carefully collected and then neutralized by adding 500 mmol/l Tris-HCL, pH 6.8.

Protein quantification was performed using the supernatants, according to the Bradford method. All the resulting supernatants were frozen at -80°C until analysis.

### Antibodies and western blotting (WB)

The following primary antibodies and working dilutions were used: rabbit polyclonal anti-APP carboxyl terminal (Sigma, 1∶5,000), rabbit polyclonal anti-ADAM 10 (Millipore, 1∶500), rabbit monoclonal anti-BACE1 (Cell Signaling, 1∶500), mouse monoclonal anti-PS1 that recognizes the PS1 amino terminal (Abcam, 1∶250), mouse monoclonal anti-AT_1_R, which recognizes the AT_1_R carboxyl terminal (Abcam, 1∶125), and mouse monoclonal anti-β actin (Sigma, 1∶1,000). Equal amounts of protein extracts (about 60 µg) were electrophoresed in sodium dodecyl sulfate-polyacrylamide gels (Bio-Rad). Proteins were transferred onto polyvinylidene difluoride membranes (Millipore). Non-specific binding was blocked with 5% non-fat dried milk in 50 mmol/l Tris-HCL, pH 7.4, containing 200 mmol/l sodium chloride and 0.5 mmol/L Tween 20 (Tris-buffered saline Tween). The blots were incubated with different primary antibodies, followed by incubation with peroxidase-conjugated anti-mouse or anti-rabbit immunoglobulins in Tris-buffered saline Tween containing 10% non-fat dried milk. Reactions were developed with an enhanced chemiluminescence system, according to the manufacturer's protocols (Pierce). The blots were then exposed to films (Kodak) for various periods of time (0.05–5 min). Data were normalized to the β-actin band.

### Fluorogenic substrate assay

Assays to evaluate secretase activity were performed according to a recent study [89]. Briefly, rat cerebral cortices were isolated and homogenized. The resulting aliquots (containing 15 µg of proteins) were centrifuged at 12,000 *g* for 10 min (Beckman). The membrane-enriched fractions were then incubated at 37°C for 30 min in 50 µl assay reaction buffer (for α-secretase, 10 mmol/L Tris-HCL, pH 6.8; for β-secretase, 50 mmol/l sodium acetate, pH 4.5; for γ-secretase, 50 mmol/l Tris-HCL, pH 6.8, 2 mmol/l EDTA, 0.25% CHAPSO) containing 10 µmol/l fluorescent conjugated peptide substrate (Calbiochem) at 37°C for 2 hr. The degree of substrate cleavage was measured as the emitted fluorescence using a reader (Tecan) at excitation/emission wavelengths of 325/393 nm (for α-secretase), 320/420 nm (for β-secretase) and 355/440 nm (for γ-secretase).

### Enzyme-linked immunosorbent assay (ELISA)

The levels of soluble Aβ_40_ and Aβ_42_ in rat cerebral cortices were detected using sandwich ELISA kits (Wako), according to the manufacturer's instructions. All samples were analyzed in duplicate.

### Statistical analysis

Data were analyzed using analysis of variance (ANOVA), followed by least significant difference *post hoc* tests, using SPSS 13.0 software (SPSS). All results were expressed as mean±standard deviation (SD).

## Supporting Information

Figure S1 **Concentration assays for Aβ_40_ and Aβ_42_ by ELISA.** n = 6 for each group. All samples were analyzed in duplicate. *p<0.05 or **p<0.01 versus the control group receiving saline infusion.
(TIF)
