# Supplementary figures and images for: Central Angiotensin II Stimulation Promotes β Amyloid Production in Sprague Dawley Rats
Source: PLoS One. 2011 Jan 28;6(1):e16037. doi: 10.1371/journal.pone.0016037 (PMC3030571; doi:10.1371/journal.pone.0016037)

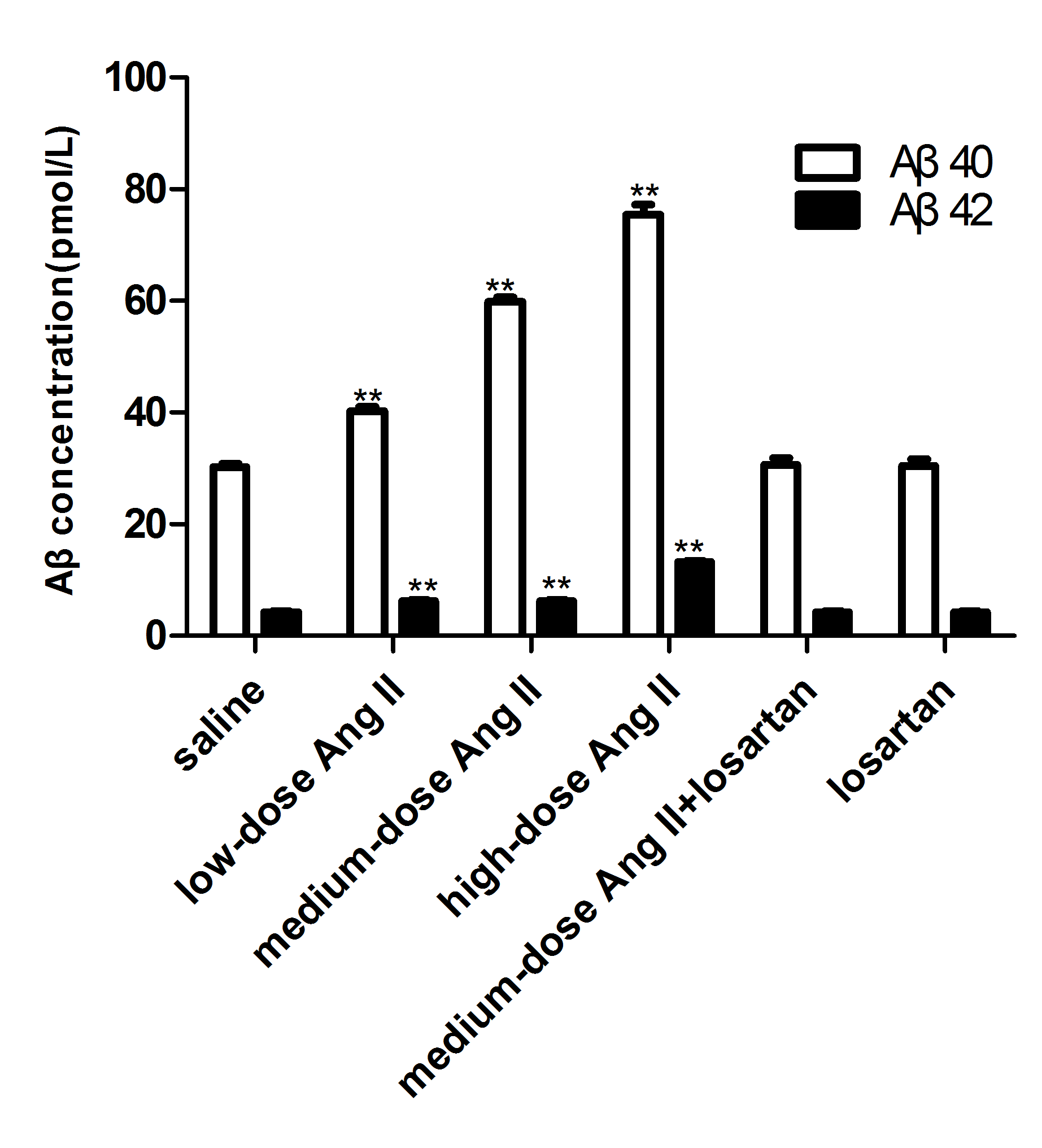

Supplement: Figure S1 — Concentration assays for Aβ40 and Aβ42 by ELISA. n = 6 for each group. All samples were analyzed in duplicate. *p<0.05 or **p<0.01 versus the control group receiving saline infusion. (TIF) [file pone.0016037.s001.tif]
